# Is cognitive behaviour therapy applicable to individuals diagnosed with bipolar depression or suboptimal mood stabilizer treatment: a secondary analysis of a large pragmatic effectiveness trial

**DOI:** 10.1186/s40345-022-00259-3

**Published:** 2022-05-03

**Authors:** Jan Scott, Richard Bentall, Peter Kinderman, Richard Morriss

**Affiliations:** 1 Institute of Neuroscience, Newcastle University, Newcastle upon Tyne, UK; 2 Department of Psychology, University of Sheffield, Cathedral Court, 1 Vicar Lane, Sheffield, S1 2LT England; 3 University of Liverpool, Liverpool, L69 3GL UK; 4 Mental Health and Cognitive Neuroscience, School of Medicine, University of Nottingham, Nottingham, UK

**Keywords:** CBT, Bipolar disorders, Depression, Sub-optimal, Treatment, Adherence, Effectiveness

## Abstract

**Background:**

Efficacy trials of medications and/or psychological interventions for bipolar disorders (BD) aim to recruit homogenous samples of patients who are euthymic and such populations show high levels of adherence to the treatments offered. This study describes a secondary analysis of a large-scale multi-centre pragmatic effectiveness randomized controlled trial (RCT) of cognitive behaviour therapy plus treatment as usual (CBT) or treatment as usual alone (TAU) and explores outcomes in individuals who were: (i) recruited in depressive episodes, or (ii) receiving suboptimal doses of or no mood stabilizers (MS).

**Methods:**

Data were extract on two separate subsamples (out of 253 RCT participants). Sample 1 comprised 67 individuals in a depressive episode (CBT: 34; TAU: 33); Sample 2 comprised 39 individuals receiving suboptimal MS treatment (CBT: 19; TAU: 20). Survival analyses (adjusted for confounding variables) were used to explore recovery in Sample 1 and relapse in Sample 2.

**Results:**

In Sample 1 (individuals with depression), Cox proportional hazards regression model revealed that the median time to recovery was significantly shorter in the CBT group (10 weeks; 95% confidence intervals (CI) 8, 17) compared to the TAU group (17 weeks; 95% CI 9, 30) [Adjusted Hazard Ratio (HR) 1.89; 95% CI 1.04, 3.4; p < 0.035]. In Sample 2 (suboptimal MS), the median time to any relapse was significantly longer in the CBT group compared to the TAU group (~ 35 versus ~ 20 weeks; Adjusted HR 2.01; 95% CI 1.01, 3.96; p < 0.05) with the difference in survival time to first depressive relapse also reaching statistical significance (X^2^ = 14.23, df 6, p 0.027).

**Conclusions:**

Adjunctive use of CBT appears to have benefits for individuals diagnosed with BD who are highly representative of the patients seen in routine clinical practice, but often excluded from efficacy RCTs. However, as this is a secondary analysis of 42% of the original RCT sample, it is important to replicate these findings in independent larger scale studies specifically designed for purpose.

**Supplementary Information:**

The online version contains supplementary material available at 10.1186/s40345-022-00259-3.

## Background

Bipolar disorders (BD) are common, severe mental disorders with high levels or mortality and morbidity. Whilst major contributors to the disease burden are the frequency of episode relapse and recurrence, it is increasingly recognised that the presence of residual symptoms, especially ongoing depression, significantly reduce quality of life (Yatham et al. 2018; Malhi et al. 2020). Overall, it is estimated that 30–60% individuals diagnosed diagnosed with BD are likely to experience a new episode onset within 12 months after an index episode and that individuals diagnosed with BD spend 50% of their lives with syndromal or sub-syndromal depressive symptoms (Judd et al. 2002, 2003; Paykel et al. 2006; Baldessarini et al. 2010). Despite some recent advances in pharmacotherapy, problems such as BD depression are rarely alleviated by medication alone (National Collaborating Centre for Mental Health 2014; Yatham et al. 2018; Malhi et al. 2020). Furthermore, the potential benefits of medications are undermined by the fact that 35–50% individuals diagnosed with BD stop their medication at least once over the course of a year (either following consultation with a clinician or against medical advice) (Scott and Pope 2002a; Levin et al. 2015). Unsurprisingly, despite advances in pharmacotherapy, most clinical practice guidelines (CPGs) for BD now discuss the need for muti-faceted approaches, and highlight the potential benefits of offering an evidence-based psychological therapy alongside any prescribed medications (e.g., National Collaborating Centre for Mental Health 2014; Yatham et al. 2018; Malhi et al. 2020).

As in any branch of medicine, CPG recommendations for BD draw heavily on evidence from meta-analyses and/or large-scale, multi-centre randomized controlled trials (RCTs) of pharmacotherapies and psychological therapies. Recommendations regarding the latter focus on four main approaches- cognitive behaviour therapy (CBT), family focused treatment (FFT), interpersonal social rhythms therapy (IPSRT) and group psychoeducation (PE) (e.g., Oud et al. 2016; Miklowitz et al. 2021). However, nearly all the findings that are quoted in contemporary CPG derive from efficacy trials that demonstrate the benefits of these adjunctive therapies in improving outcomes in homogeneous samples of individuals diagnosed with BD who were recruited during euthymia (i.e., do not have clinically significant levels of depressive symptoms) and/or at a time when they are adherent with prescribed mood stabilizers (MS). These efficacy RCTs are very important and the eligibility criteria minimize confounding and biases (e.g., by recruiting homogeneous samples), which in turn maximizes opportunities to detect treatment effects. However, the consequences of this sampling strategy are the potential loss of external validity and generalizability (Scott 2008; Etain et al. 2018). This was demonstrated clearly by Hoertel et al. (2013) who reported that more than 50% individuals diagnosed with BD are excluded from these efficacy RCTs (58–64% for depression trials; 56% for hypo/mania trials). The excluded cases were those that were least likely to respond to the experimental treatments or interventions, and/or were less likely to adhere with pharmacotherapy, meaning that there are several clinically relevant questions about the use of psychological interventions to improve outcomes (response or recovery) for individuals diagnosed with BD that still need to be addressed (Etain et al. 2018). For example, unresolved questions regarding the management of BD include how best to overcome BD depression and how to improve real world outcomes, given the high rates of suboptimal adherence with medications (McIntyre et al. 2020). These two questions are also interesting from the perspective of the putative benefits of psychological therapies and the clinical need to commence therapies in a timely manner for individual patients seen in real world settings. So, clinicians need to know whether adjunctive psychological interventions improve clinical outcomes if the therapy commences is offered to individuals diagnosed with BD during a depressive episode or during a period of low- or non-adherence with a recognized MS. We briefly review the evidence regarding these issues below.

Whilst there is some evidence for the impact of psychological interventions on depressive episodes in individuals diagnosed with BD this derives from relatively small-scale trials many of which were rated as low quality by independent assessors (Yilmaz et al. 2022). The most widely quoted evidence of efficacy derives from the Systematic Treatment Enhancement Program for Bipolar Disorder (STEP-BD) study. This incorporated two multi-site RCTs that examined the effects of different medication regimes and different psychological therapies on acutely depression in individuals diagnosed with BD I and II (Sachs et al. 2007; Miklowitz et al. 2007). The 12-month psychological therapy RCT, compared three intensive therapy models (30 sessions of FFT, Group CBT, or IPSRT) with a 3-session collaborative care intervention (Miklowitz et al. 2007). The trial demonstrated that individuals receiving a 9-month course of therapy had significantly higher recovery rates (64% versus 52%) and a reduced time to recovery from acute BD depression (median 113 versus 146 days) compared to the collaborative care condition. However, 80% (236 of the 293) of participants in the psychological treatment RCT were simultaneously participating in the pharmacotherapy RCT. Out of 75 individuals randomized to CBT only 18 (24%) were recruited solely to the therapy RCT [along with 15% (4/26) FFT and 17% (11/62) IPSRT participants]. Thus, there are still some gaps in our knowledge regarding the benefits of adjunctive therapy compared with treatment as usual (TAU; where TAU is defined as the prescription of medications with or without other interventions that reflects locally accepted treatment practices for individuals diagnosed with BD) (Yilmaz et al. 2022). Likewise, there is limited data on the use of CBT for individuals diagnosed with BD who are not being prescribed and/or decline to take medication. Given the obvious ethical concerns about undertaking research in this population (and/or e.g., withdrawing MS medication to participate in a RCT), the available publications comprise largely of small ‘N’ case series of individuals diagnosed with BD-II (and/or cyclothymia) or of naturalistic case reports about individuals who were not taking MS (Reilly-Harrington et al. 2007; Swartz et al. 2018; Vallarino et al. 2015).

An alternative option for exploring these outstanding issues about the use of psychological interventions in BD is to undertake secondary analyses of an existing RCT dataset. We previously reported the findings of a large scale, pragmatic multi-centre RCT that examined whether CBT added to usual treatment was more effective than TAU alone in reducing recurrence rates in a heterogeneous, but clinically representative sample of 253 individuals with severe and recurrent BD (Scott et al. 2006). The primary analyses did not find any statistically significant differences in number of or time to first relapse. However, a post-hoc analysis found that those who had experienced fewer prior BD episodes were more likely to benefit from adjunctive therapy. Of course, a major issue for the trial was the ‘noise’ created by the minimal exclusion criteria and subsequent clinical heterogeneity of the sample. Whilst some experts viewed these as methodological weaknesses (Lam 2006), it means we are now able to identify 67 individuals (26.5% of the original RCT sample) who were in a current depressive episode at the time of randomization and a separate subgroup of 39 individuals (15% of the sample) who, when interviewed by independent trained assessors (masked to group allocation), were found to be receiving suboptimal MS treatment. This paper assesses the 18-month outcomes of these individuals who are highly representative of patients diagnosed with BD seen in day-to-day clinical practice, but who are usually excluded from RCTs of pharmacotherapy and psychotherapies.

## Methodology

Full details of the methodology (including the trial flow chart, etc.), are described elsewhere (Scott et al. 2006). Additional file 1: Table S1 summarizes key demographic and clinical characteristics of the trial participants. In this section we summarize the key elements of the original RCT and provide details of the current study.

### Trial design

Ethical approval was obtained from the UK North East Multi-Centre Research Ethics Committee to undertake a 5-centre multi-centre, pragmatic, randomised controlled trial of adjunctive CBT compared with treatment as usual (TAU) in BD employing independent blind assessments of progress and outcome over 18 months.

### Sample

For the main trial, we recruited 253 participants who were willing and able to give written informed consent and who also met the following inclusion criteria: (a) aged ≥ 18 years; (b) diagnosis of BD I or II according to DSM-IV criteria (American Psychiatric Association 1994); (c) history of two or more episodes of illness meeting DSM IV criteria for mania, hypomania, major depressive disorder or mixed affective disorder, one of which must have been within 12 months prior to recruitment; and (d) in contact with mental health services within the last six months. Exclusion criteria were: (a) rapid cycling bipolar disorder (defined as > 4 episodes of mania and depression alternating with less than one month in between in the last year); (b) BD secondary to an organic cause; (c) evidence of severe borderline personality disorder with suicidal ideation or intent in the past 3 months (as brief CBT or usual treatment alone may be unethical); (d) continuous illicit substance misuse resulting in uncertain primary diagnosis; (e) currently meeting DSM IV criteria for mania (although these individuals could be included when symptoms had subsided to reach criteria for hypomania); and (f) current exposure to a systematic psychological treatment specifically aimed at managing BD.

The Independent Trials and Biostatistics Office (Manchester, UK) randomized all trial participants using a minimisation algorithm that balanced across number of previous episodes, current mental state and trial centre. It should be noted that this algorithm would lead to a similar number of individuals with a depressive episode being allocated to each group. However, the algorithm did not specifically consider medication adherence or suboptimal treatment. One hundred and 27 individuals were allocated to TAU alone and 126 to CBT.

For the current study, we extracted two independent sample from the dataset using the following additional eligibility criteria-

#### Sample 1

Eligible individuals met DSM IV criteria for a current major depressive episode. The total N was 67 and comprised 34 participants allocated to CBT and 33 to TAU.

#### Sample 2

Individuals were eligible for inclusion if they did not meet criteria for a current full-threshold BD episode (this criterion was included primarily to ensure there was no overlap with Sample 1) and were identified as receiving sub-optimal MS. The latter included those who were not currently being prescribed a recognized MS, were not taking another medication (such as an antipsychotic) that was prescribed specifically for the purpose of mood stabilization, and/or they met criteria for low adherence or were non-adherent with a prescribed MS [low adherence defined as < 50%, as assessed using the Tablet Routines Questionnaire (TRQ)] (Adams and Scott 2000; Scott and Pope 2002a, b). The total N was 39 and comprised 19 participants allocated to CBT and 20 to TAU.

### Outcome measures

Trained research assistants masked to treatment condition conducted all assessment interviews immediately prior to randomization and then face-to-face interviews every eight weeks for 72 weeks. Inter-rater reliability was high and was monitored throughout the RCT. For the purposes of the current study we report: (a) baseline assessment information- including demography, clinical history and current treatments, clinical ratings on the 17-item Hamilton Rating Scale for Depression (HRSD; Hamilton 1960) and the Bech-Rafaelson Mania Scale (BRMS; Bech et al. 1978) and medication adherence (using the TRQ); and (b) clinical outcome- as the goal is to compare outcomes in the CBT and TAU groups, we report two sets of outcome data.

For Sample 1, we report the proportion of individuals who met criteria for recovery from a current depressive episode (defined as HRSD ≤ 8 for 8 consecutive weeks) (Paykel et al. 2006) and median time to recovery.

For Sample 2, we report the proportion of individuals who experienced a new episode of BD, defined as the onset of a full-threshold episode of BD of any polarity that met DSM-IV criteria (i.e., manic, hypomanic, mixed or depressive) and the time to episode onset.

### The interventions

All individuals were offered TAU (clinical management and medication), which was regarded as the control condition, and 50% of the sample was randomly allocated to receive CBT also (which was considered the active therapy).

#### TAU

This was administered to all participants by their usual psychiatric treatment team. It included prescription of medications and contact with key mental health professionals with whatever frequency considered appropriate. Clinicians were specifically asked not to introduce any form of systematic psychotherapy for bipolar disorder for the duration of the study, so formal psychoeducational interventions were excluded from the clinical support, but there were no other treatment constraints.

#### CBT

This comprised 20 sessions of CBT over six months (weekly till session 15, then gradually reducing in frequency). Two ‘booster sessions’ were offered to patients (at weeks 32 and 38) to review the skills and techniques learned. The CBT approach is described elsewhere but was based on Beck’s model and is similar to the formulation based approaches described for other severe mental disorders, with specific elements added to target problems in BD and to aid relapse prevention (for a description see Scott et al. 2001; Scott 2022). Further, the model included strategies to ameliorate acute depressive symptoms, mood instability and to enhance medication adherence.

### Statistical analysis

All analyses were undertaken using SPSS (version 27). There were small amounts of random missing data (e.g., single item ratings from assessments or one rating scale score from a single follow-up appointment), as such we replaced these missing values with sample means. All statistical significance was set at p < 0.05.

The interval in weeks from randomisation to recovery or from randomization to new episode onset were analysed using Kaplan Meier curves with significance tests based on the Cox proportional hazards regression model. Treatment group, number of previous BD episodes (categorized as above or below the group medians for depression and/or for mania for each sub-sample), were included as covariates, along with sex, and psychiatric comorbidity. For Sample 1, baseline HRSD score was included also as a covariate. For Sample 2, separate analyses were conducted for any relapse and then for depressive relapses (numbers for other relapses were too low to justify further statistical analysis).

## Results

### Sample 1

As shown in Table 1, the CBT and TAU groups did not differ significantly on any baseline clinical or demographic features. The mean age of the participants was about 40 years, about 65% were female (44 of 67 individuals); 65 individuals met DSM IV criteria for BD I. The HRSD scores indicated individuals had moderately severe levels of depressive symptoms. Regarding treatment regimes and adherence, there was non-significant trend for more individuals in the TAU (18%) compared with the CBT group (9%) to be prescribed ≥ 3 medications. Adeherence with MS averaged about 80% across both groups; individuals allocated to CBT attended about 75% therapy sessions offered (median 18).

**Table 1:** Key characteristics of individuals with an index depressive episode

	TAU (n = 33)	CBT (n = 34)
Mean age (SD)	39.67 11.41)	41.43 (12.85)
Female	20 (61%)	24 (70%)
Current comorbid mental disorder	17 (52%)	16 (47%)
Current substance misuse	2 (6%)	3 (9%)
Median HRSD score (IQR)	16 (10, 23)	15 (9, 21)
Median BRMS score (IQR)	1 (0, 2)	1 (0,2)
Median number of episodes (IQR)		
Depression	7.5 (2–13)	8 (2–15)
Mania/hypomania/mixed states	5 (1–7)	4 (1–6)
Current medication		
≥ 3 Medications	6 (18%)	3 (9%)
Lithium and/or anti-convulsant	27 (82%)	27 (79%)
Anti-psychotic	18 (55%)	21 (62%)
Anti-depressant	26 (64%)	25 (74%)
Current adherence level > 75%	31 (94%)	30 (88%)

Adherence level measured using the tablet routines questionnaire

*SD* standard deviation, *IQR* inter-quartile range

**Fig. 1:**
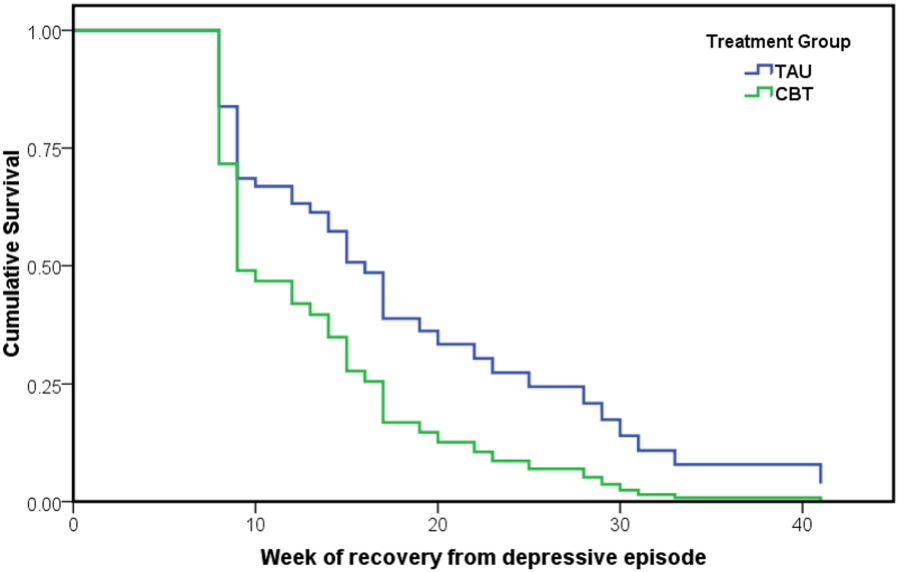
Survival curves of time to recovery from index depressive episode according to group (Cox regression analysis; see text for co-variates)

Fifty-eight individuals (86%) met criteria for recovery during the follow-up period, comprising 27 (82%) TAU recipients and 31 (91%) CBT recipients (see Additional file 1: Tables S2). As shown in Fig. 1, time to recovery from depression, was significantly longer in the TAU group [median 17 weeks; 95% confidence intervals (CI) 9, 30] compared to the CBT group (median 10 weeks; 95% CI 8, 17), and Cox regression analysis demonstrated that the most important predictor of time to recovery was treatment group [Adjusted Hazard Ratio (HR) 1.89; 95% CI 1.04, 3.4; p < 0.035] (see Additional file 1: Table S3).

### Sample 2

As shown in Table 2, the two groups did not differ significantly on any clinical or demographic features. The mean age of the participants was about 40 years, nearly all (n = 37) met DSM IV criteria for BD I, and > 70% were female. Three individuals were not taking any medication at the time of randomization, whilst a small proportion of individuals (CBT = 4; TAU = 2) were taking other medications or ‘alternative’ treatments (this included drugs not usually licensed for BD, or used for co-occurring symptoms e.g., pregabalin for anxiety; or over the counter alternatives e.g., St John’s Wort). In the 36 individuals receiving any current medication, just over 50% reported low adherence or non-adherence for prescribed medications. Therapist records showed that those allocated to CBT attended about 66% therapy sessions offered (median 17).

**Table 2:** Key characteristics of individuals with suboptimal or no mood stabilizer treatment

	TAU (n = 20)	CBT (n = 19)
Mean age (SD)	43.1 (10.97)	41. 21 (11.3)
Female	14 (70%)	14 (74%)
Current comorbid mental disorder	6 (30%)	10 (53%)
Current substance misuse	2 (10%)	1 (5%)
Median HRSD score (IQR)	6 (3, 9)	7 (3, 10)
Median BRMS score (IQR)	2 (0, 3)	2, (1, 3)
Median number of episodes:		
Major depression (IQR)	3.8 (1–9)	4 (1–10)
Mania/hypomania/mixed (IQR)	3 (2–6)	2.9 (2–6)
Current medication		
None	2 (10%)	1 (5%)
Benzodiazepine monotherapy	0	1 (5%)
Anti-depressant monotherapy	6 (30%)	5 (26%)
Other medication	2 (10%)	4 (21%)
Current adherence level < 50%	11 (55%)	11 (58%)

Adherence level measured using the tablet routines questionnaire

*SD* standard deviation, *IQR* inter-quartile range

As shown in Table 3, 25 individuals (64% sample) had a BD relapse during the follow-up period, comprising 10 (53%) CBT and 15 (75%) TAU recipients. Most first relapses (n = 19) were depressive comprising seven (37%) CBT and 12 (60%) TAU, whilst six were manic, hypomanic or mixed (CBT = 3; TAU = 3) (see Additional file 1: Figs. S1 and S2). The median time to any relapse was longer in the CBT group compared to the TAU group (~ 35 versus ~ 20 weeks) with the difference in survival time to first depressive relapse reaching statistical significance (CBT ~ 42 versus TAU ~ 21 weeks; p = 0.027). Cox regression analysis demonstrated that treatment group was the significant predictor of time to relapse (Adjusted HR 2.01; 95% CI 1.01, 3.96; p < 0.05) (see Additional file 1: Table S3).

**Table 3:** Time to any relapse and time to depressive relapse

	N patients	Relapse		CBT vs TAU^a^
		N (%) events	Median time in weeks (inter-quartile range)	
Any relapse				
TAU	20	15 (75%)	20.3 (5.45–34.78)	X^2^ = 11.37 df 6
CBT	19	10 (53%)	35.7 (11.47–58.53)	p = 0.07
1st depressive relapse				
TAU	20	12 (60%)	21.5 (8.45–39.6)	X^2^ = 14.23 df 6
CBT	19	7 (37%)	42.2 (25.2)^b^	p = 0.027

^a^Co-variates are listed in methods

^b^Full inter-quartile range cannot be shown as < 50% CBT cases experienced a depressive relapse

## Discussion

This study represents a secondary analysis of two subsamples of individuals diagnosed with BD and uses data from a previously published pragmatic effectiveness trial. Sample 1 (individuals with a current depressive episode) comprised about a quarter of the original trial sample, whilst Sample 2 (suboptimal or no MS treatment) comprised about 15%. Although both subsamples are similar in socio-demographic and baseline clinical characteristics (apart from depression and adherence etc.) to the original sample, we emphasize the importance of considering the findings in the context of these weaknesses and potential limitations. Three major issues are worth re-iterating here. First, the original pragmatic effectiveness RCT used a minimization algorithm that ensured that there was a balanced allocation of individuals with depression across the CBT and TAU groups, but did not consider suboptimal MS. Whilst the samples we analysed do appear similar on most key variables, secondary analyses, especially of subpopulations extracted from larger RCTs must always be treated with caution. This issue is important to emphasize as the main finding from the original study (intent to treat analysis of CBT versus TAU) did not identify significant differences in outcome. Second, the original study design did not include provision for the sub-analyses we report. This is especially important with regards to the statistical power of our analyses, especially in sample 2. Third, although the original RCT was not focused on BD-I, the majority of the subsamples were comprised of this subtype, so we cannot be certain how the current findings would apply to BD-II. Having noted these concerns, we now discuss our findings.

In the present study, about 80% of individuals in an index depressive episode recovered during the 18 months of follow-up. About 9% more CBT as compared to TAU cases met recovery criteria during the follow-up period and time to recovery was significantly shorter, by about 7 weeks, in those receiving CBT compared to TAU alone (median 10 versus 17 weeks). In the STEP-BD study, the overall recovery rate was lower (~ 55%), but the follow-up period was for one year and the 30 sessions of CBT were delivered in a group rather than individual format (Miklowitz et al. 2007). Interestingly, the between group difference in recovery rates (difference 8.5%) and median time to recovery (median CBT vs. control =  ~ 16 vs. ~ 21 weeks) were very similar to the present study.. These findings are noteworthy given those of a recent meta-analysis that suggested that there was significant effect on symptoms of BD depression for CBT and dialectical behaviour therapy but not for psychoeducation, mindfulness-based therapy, FFT or IPSRT (Yilmaz et al. 2022). Similarly, a more sophisticated network component meta-analysis of the benefits of adding psychological therapies to TAU in the management of BD indicated that CBT was associated with better depression outcomes than FFT or IPSRT (Miklowitz et al.^ 2021^). Added to substantial evidence that CBT for unipolar depression is a highly efficacious intervention, we believe the current findings should encourage a larger scale independent study of CBT for acute BD depression—a condition that is has provided refractory to most widely used antidepressants.

Given the smaller sample of individuals with suboptimal MS treatment and the lower statistical power of this post-hoc analysis, it is unsurprising that the between group comparison of relapse rates produced more mixed findings. The survival analyses suggest a significantly reduced risk of depressive relapse if CBT is offered to these individuals (median time to relapse with CBT × 2 that of TAU). The subsample comprises some individuals not taking or not prescribed a MS as well as those with low levels of adherence, but it was not possible to undertake further within group explorations. Also, whilst we deliberately chose a definition of low MS adherence that is well below reported thresholds for ‘effective prophylaxis (75% adherence with a MS is usually regarded as the minimum required), there is no international consensus and relatively limited research on the validity of these cut-offs (Scott and Pope 2002a, b; Bauer et al. 2010; Velligan et al. 2010; Levin et al. 2015). Despite this, the fact remains that 54% of individuals (3 not on any medications; 1 on benzodiazepines alone; 11 on antidepressants alone; 6 on other medications) were either not taking medication or were receiving medication regimes that are not usually regarded as adequate, not recommended for, or specifically not supported in clinical guidelines on the treatment of BD (Gomes et al. 2021). Of course, there may be a number of reasons for these treatment approaches, but clinical and research studies suggest that these sub-optimal treatment regimes are commonplace. Overall, individuals in the CBT group in Sample 2 attended about two thirds of the sessions offered to them (which is similar to day-to-day settings). However, individuals with low or non-adherence with medication did not necessarily fail to adherence with CBT. Thus, the use of CBT in this population offers an insight into the likely real-world benefit of adjunctive psychological treatment when offered in less favourable circumstances. It is possible to speculate that some of these individuals were ambivalent about (or even against) the use of pharmacotherapy but were more accepting of a psychological approach. Our dataset is too small to attempt mediation analyses, but it is reported that psychological interventions lead to improved medication adherence in individuals diagnosed with BD (Colom et al. 2003a, b; Etain et al. 2018). However, enhancing adherence does not fully account for the gains attributed to psychological interventions, and some but not all research demonstrates reductions in relapses in those receiving psychoeducation who are already highly adherent to medication (Colom et al. 2005; Morriss et al. 2016) and that change in attitudes towards BD and enhanced self-management may be as, if not more critical components (Miklowitz et al. 2021). In sum, our findings offer tentative support for the possibility that, if it is the only realistic option available, there could be some benefits to providing psychological monotherapy in BD. However, we emphasize that the CBT we provided was a manualized intervention undertaken by expert therapists who participated in intensive training and received ongoing supervision, i.e., this study offers no evidence that the provision of generic models of CBT by therapists without supervision or peer support would ever be appropriate for individuals diagnosed with BD who are taking suboptimal medication treatments.

## Conclusions

This secondary analysis of a large-scale trial of CBT for severe and recurrent BD offers evidence of the particular utility of adjunctive CBT in treating depressive episodes leading to earlier recovery and delaying depressive relapses. The inclusion of individuals in a current depressive episode or with suboptimal MS treatment means the findings are useful for clinicians working with individuals diagnosed with BD. The fact that the benefits of CBT for BD mirror the findings for unipolar disorders (CBT can be used in individuals whether or not they are currently experiencing an acute episode and can also be used in those who do not respond to or do not receive adequate antidepressant treatment) may also shed light on mechanisms of action of CBT, and thus generate research questions. Lastly, this study also provides data that could be used to inform sample size and power calculations for future effectiveness RCTs.

## Supplementary Information

**Additional file 1: Table S1.** Baseline characteristics of original RCT sample (n = 253). **Table S2.** Median time to recovery from index depressive episode (Kaplan Meier). **Table S3.** Cox regression analysis showing predictors of time to recovery from index depressive episode. **Figure S1.** Cumulative time to any recurrence in individuals with no or suboptimal mood stabilizer treatment. **Figure S2.** Cumulative time to depressive recurrence in individuals with no or suboptimal mood stabilizer treatment.

## Data Availability

The authors confirm that the summary data for all variables supporting the findings of this study are included within the article and its Additional file. The raw data can be made available via the corresponding author upon written application and subject to the approval of the relevant research and ethics committees.

## Abbreviations

BD: Bipolar disorders
CBT: Cognitive behaviour therapy
CPG: Clinical practice guideline
DSM: Diagnostic and statistical manual
FFT: Family focused treatment
HR: Hazard ratio
PE: Group psychoeducation
IPSRT: Interpersonal social rhythms therapy
MS: Mood stabilizer
RCT: Randomized controlled trial
STEP-BD: Systematic treatment enhancement program for bipolar disorder TAU treatment as usual
UK: United Kingdom
