# Supplementary material for: Is cognitive behaviour therapy applicable to individuals diagnosed with bipolar depression or suboptimal mood stabilizer treatment: a secondary analysis of a large pragmatic effectiveness trial
Source: Int J Bipolar Disord. 2022 May 3;10:13. doi: 10.1186/s40345-022-00259-3 (PMC9061901; doi:10.1186/s40345-022-00259-3)
Supplement: Supplementary file 1 — Additional file 1: Table S1. Baseline characteristics of original RCT sample (n = 253). Table S2. Median time to recovery from index depressive episode (Kaplan Meier). Table S3. Cox regression analysis showing predictors of time to recovery from index depressive episode. Figure S1. Cumulative time to any recurrence in individuals with no or suboptimal mood stabilizer treatment. Figure S2. Cumulative time to depressive recurrence in individuals with no or suboptimal mood stabilizer treatment. [file 40345_2022_259_MOESM1_ESM.docx]

**APPENDIX**

**Additional Tables**

**Table S1: Baseline characteristics of original RCT sample (n=253)**

| **Characteristic** | **Number (%)** |
| --- | --- |
| Female Sex | 164 (65) |
| Bipolar I disorder | 238 (94) |
| Bipolar II disorder | 15 (6) |
| In episode at baseline | 88 (35) |
| Current anxiety or eating disorder | 58 (23) |
| Current substance use disorder | 26 (11) |
| Borderline or antisocial personality disorder | 9 (7) |
| On Lithium or Anti-convulsant mood stabiliser | 213 (84) |
| On antidepressants | 109 (43) |
| On antipsychotics | 127 (50) |
| On benzodiazepines | 46 (18) |

**Table S2: Median time to recovery from index depressive episode (Kaplan Meier)**

| **Treatment Group** | **Number Recovering (%)** | **Time to Recovery in Weeks** | | | | | |
| --- | --- | --- | --- | --- | --- | --- | --- |
|  |  | **Median** | | **25%** | | **75%** | |
|  |  | **Estimate** | **Std. Error** | **Estimate** | **Std. Error** | **Estimate** | **Std. Error** |
| **Treatment as Usual** | 27 (82%) | 17 | 2.45 | 9 | .48 | 30 | 4.97 |
| **Cognitive Behaviour Therapy** | 31 (91%) | 10 | 1.61 | 8 | .21 | 17 | 3.89 |

**Table S3: Cox regression analysis showing predictors of time to recovery from index depressive episode**

|  | **Wald** | **df** | **Sig.** | **Exp(B)** | **95.0% CI for Exp(B)** | | **Chi-squared** |
| --- | --- | --- | --- | --- | --- | --- | --- |
|  |  |  |  |  | **Lower** | **Upper** |  |
| **Treatment Group** | 4.46 | 1 | .035 | 1.89 | 1.04 | 3.40 | 13.26  df 2  p=0.001 |
| **Severity of Depression**  **(HRSD score)** | 9.18 | 1 | .04 | .90 | .84 | .96 |  |

**Additional Figures**

**Figure S1: Cumulative time to any recurrence in individuals with no or suboptimal mood stabilizer treatment**

+ Treatment as Usual

+ CBT

**Figure S2: Cumulative time to depressive recurrence in individuals with no or suboptimal mood stabilizer treatment**

+ Treatment as Usual

+ CBT
